# *Coxiella burnetii* Antibody Prevalence and Risk Factors of Infection in the Human Population of Estonia

**DOI:** 10.3390/microorganisms7120629

**Published:** 2019-11-29

**Authors:** Kädi Neare, Marilin Janson, Pirje Hütt, Brian Lassen, Arvo Viltrop

**Affiliations:** 1 Institute of Veterinary Medicine and Animal Sciences, Estonian University of Life Sciences, 51006 Tartu, Estonia; marilin.janson@emu.ee (M.J.); arvo.viltrop@emu.ee (A.V.); 2 OÜ Tartu Kesklinna Perearstikeskus, 51005 Tartu, Estonia; pirje.hutt@tlpak.ee or or; 3 OÜ Perearst Marje Koha, 50410 Tartu, Estonia; 4 Department of Microbiology, University of Tartu, 50411 Tartu, Estonia; 5 Department of Veterinary Disease Biology, University of Copenhagen, 1870 Fredriksberg C, Denmark; brian.lassen@gmail.com

**Keywords:** North–Eastern Europe, coxiellosis, public health, people, occupational risk, epidemiology

## Abstract

Q fever is an emerging health problem in both humans and animals. To estimate the prevalence of *Coxiella burnetii* (*C. burnetii*) antibodies in the Estonian population, we analyzed plasma samples from 1000 individuals representing the general population and 556 individual serum samples from five population groups potentially at a higher risk (veterinary professionals, dairy cattle, beef cattle, and small ruminant stockbreeders and hunters). Additionally, 118 dairy cow bulk tank milk samples were analyzed to establish the infection status of the dairy cattle herds and the participating dairy cattle keepers. Questionnaires were used to find the potential risk factors of exposure. The effects of different variables were evaluated using binary logistic regression analysis and mixed-effects logistic analysis. The prevalence in veterinary professionals (9.62%; *p* = 0.003) and dairy cattle farmers (7.73%; *p* = 0.047) was significantly higher than in the general population (3.9%). Contact with production animals in veterinary practice and being a dairy stockbreeder in *C. burnetii* positive farms were risk factors for testing *C. burnetii* seropositive (*p* = 0.038 and *p* = 0.019, respectively). Results suggest that *C. burnetii* is present in Estonia and the increased risk of infection in humans is associated with farm animal contact.

## 1. Introduction

Q fever is a widespread zoonosis with pandemic potential caused by the intracellular bacterial pathogen *Coxiella burnetii* (*C. burnetii*) [1]. In some regions of the world, especially in islands such as American Samoa and Tahiti, the bacteria have not yet been detected, either because of its absence or its sporadic presence [1,2,3]. The estimates for *C. burnetii* antibody-positive humans in Europe varies between geographical regions and population groups, ranging from 2.4% in the Netherlands (2006–2007) [4] and 5.35% in France (1995) [5] to 38% in Bulgaria (1995) [6]. The estimate ranges from 31.12% of Polish to 84% of Danish and Dutch persons having contact with ruminants [7,8,9,10,11]. During 2013–2016 reports of human cases in Europe in total increased from year to year but decreased in 2017. However, in some countries, such as Bulgaria and Spain, the number of reported cases increased. Low numbers might not reflect the real situation, as some European Union/European Economic Area countries do not report the presence of *C. burnetii* infection [12]. Furthermore, these prevalence estimates should be compared with caution as different approaches to determine seropositivity for *Coxiella burnetii* infection have been used in these studies. In most studies, indirect immunofluorescent assay (IFA) [2,3,7,11] or enzyme-linked immunosorbent assay (ELISA) [4,13] has been used, and less frequently the complement fixation test (CFT) [13]. The IFA has been shown to be the most sensitive and specific test for *C. burnetii* antibody detection, but since IFA testing is more laborious, combining ELISA as a primary screening test and IFA as a confirmatory test has been suggested for large-scale population studies [14].

Domestic ruminants are considered one of the main infection sources for humans [13,15,16,17,18,19,20]. The infection transmits to humans mainly through the inhalation of aerosols containing *C. burnetii* [13,15,19,21] or via direct contact with infected animals [22,23]. In lactating animals, the bacterium is excreted in the milk [24,25,26], and consuming unpasteurized goat’s milk, cow’s milk, or raw milk products is a risk factor for acquiring the infection [18,23,27]. *Coxiella burnetii* produces small spore-like forms [28] that enable the pathogen to survive in the environment for long periods of time [29,30]. Some environmental and meteorological conditions increase the risk of infection, such as higher wind speeds, soil, and landscapes being more sensitive to wind erosion and low rainfall [15,21,31]. A number of animal species, including pets [32,33,34,35], horses [36,37], birds [38], wildlife, and arthropods (mainly ticks) [39,40,41,42], might also play a role in spreading *C. burnetii*.

The main risk factors for human infection are direct or indirect contact with parturient ruminants [13,19,23,43] and traveling or working in areas where the pathogen is endemic [44,45,46]. Farm and abattoir personnel, along with veterinarians, are occupations considered to be at a higher risk of getting the infection due to the contact with animals [8,9,10,18,47,48]. The course of the infection depends on the genetic differences, origin, and virulence of the strain [49,50] and varies from an asymptomatic or flu-like disease to being fatal [13,23,51]. Men are reported to be more likely to be found positive for antibodies against *C. burnetii* or to be diagnosed with Q fever [9,10,43,47,52]. Excretion of the bacterium via milk and faeces has been reported in humans [39].

So far, there has been little information on Q fever’s distribution and risk factors in North-Eastern Europe [12,53]. The aim of the current study was to estimate the prevalence of *C. burnetii* antibodies in the general adult population and risk groups in Estonia and to identify the risk factors of a *C. burnetii* infection.

## 2. Materials and Methods

Blood samples were collected from each study group (the general population and risk groups) separately using a cross-sectional design. The risk groups were veterinarians, their assistants, and final year veterinary students, considered as a single group and called hereafter ‘veterinary professionals’, dairy cattle; beef cattle, and small ruminant keepers and hunters. The minimum sample size was calculated for every study group to estimate the apparent prevalence with a 95% confidence level, assuming a 20% expected prevalence and allowing for a +/−5% error of the estimate, considering the total number of individuals in each group in Estonia. The expected prevalence for sample size calculations was set based on the results of previous population studies from the Baltic Sea region [7,18,54]. The desired minimal sample size was 212 for veterinary professionals and 246 for every other study group. The sample size calculations were performed with the EpiTools epidemiological calculator (2012) [55].

Plasma samples from the general Estonian adult population (the reference group) were obtained from the biobank of the Estonian Genome Centre (EGC) [56], which at the time of study had approximately 50,000 blood plasma samples from volunteer donors all over the country collected between 2002–2011. A random sample of 1000 individuals was selected from the collection using a random number generator. The sample was stratified by county, taking the population density and gender balance of each county into account. The demographical data used for stratification were received from Statistics Estonia [57]. Plasma samples were stored at −20 °C until analysis.

The convenience sample from the five potential risk groups was collected on a voluntary basis during 2012–2014 as follows:

- Veterinary professionals (*n* = 158), including veterinary practitioners (*n* = 115), veterinary technicians (*n* = 15), final year veterinary students (*n* = 26), and laboratory veterinarians (*n* = 2) during the annual national veterinary conference in October 2012.
- Dairy cattle keepers (*n* = 193), beef cattle keepers (*n* = 52), and small ruminant keepers (*n* = 63) during “information days” organized by the Estonian Livestock Performance Recording Ltd. from 2013–2014.
- Hunters (*n* = 144) during the annual meeting in July 2013.

After collection and clotting, each blood sample was centrifuged at 1500 rpm for 10 min. A total of 1 mL of serum was then separated by pipetting, tagged, and stored at −20 °C until testing.

10–500 mL of bulk tank milk samples (*n* = 117) were collected from the dairy cattle herds of the keepers participating in the study to determine the herds’ *C. burnetii* status. Bulk tank milk samples were taken by the animal keepers and stored in a refrigerator until submitted to investigators within 24 h. Bulk tank milk samples were thoroughly mixed using a vortex mixer. A total of 1 mL of milk was then separated by pipetting into 1 mL centrifuge tubes, labelled, and stored at −20 °C until analysis.

Questionnaires were used to obtain information about the possible risk factors. For the general population, the questionnaire data collected by the EGC from the donors was used. Participants from the risk groups completed specially designed questionnaires after the blood was collected. The information included: age, gender, habitation, and educational level, as well as occupation, nature of the work, and occupational environment (farmers and veterinary professionals), eating habits, activities, pets, and occupational and personal hygiene and protection. Information regarding the proportions of the different species of handled animal patients was obtained from the veterinary professionals. Although two different questionnaires were used for general population and risk groups, the questions asked in both questionnaires overlapped significantly, allowing us to make comparisons.

All human samples were examined for *C. burnetii* antibodies with a *Coxiella burnetii* Phase 2 IgG ELISA test kit (Virion\Serion, Würzburg, Germany). Samples testing positive, and those testing negative but with optical density (OD) values of 0.38 and above (*n* = 84), were confirmed with a Q Fever IFA IgG test kit (Focus Diagnostics, Cypress, CA, USA) using single 1:16 dilution. The two-tier approach was used considering available resources, since ELISA tests are cheaper and less labor-intensive for screening. The more sensitive and specific IFA was used to detect possible false negative and false positive ELISA test results. Bulk tank milk samples were analyzed using a PrioCHECK Ruminant Q Fever Ab Plate Kit (Thermo Fisher Scientific, Waltham, MA, USA). All tests were performed and evaluated according to the instructions of the manufacturers.

Prior to data analysis, all laboratory results were anonymously linked to the questionnaire data. Data regarding two veterinary professionals were excluded, because the persons lived abroad. Data of 12 dairy cattle keepers, 11 beef cattle keepers, 19 small ruminant keepers, and 10 hunters were excluded because the questionnaires were not returned.

Veterinary professionals were further categorized to farm animal professionals and other professionals based on the proportion of farm animal patients they handled (see Figure S1 in Supplementary Materials). They were considered to be farm animal professionals if 50% or more of their patients were horses and/or production animals (domestic ruminants, pigs, chickens and other farm birds, farm rabbits, and fur animals), and “other professionals” if the proportion of the farm animal patients was below 50%.

The EpiTools epidemiological calculator (2019) was used to calculate seroprevalences and confidence intervals (by Wilson’s method) [55]. The free software R version 3.5.2 [58] was used to evaluate the effect of the variables. Preliminary correlation testing and binomial logistic regression analysis were carried out with package Rcmdr, and package lme4 was used for mixed-effects logistic regression analysis. All of the information was used in the preliminary correlation testing, which was performed using a chi-squared test, Pearson’s correlation test, and univariable binomial logistic regression analysis. All variables with a *p*-value of <0.6 were considered in model building; other variables were excluded from further analysis.

The first model was developed to compare the prevalence within the general population and risk groups. The model was built by forward inclusion of variables, starting from the variable of main interest. While adding new variables, next to *p*-values, the Akaike information criterion (AIC) was used to monitor the model fit.

Two models were developed to evaluate the risk factors for infection (antibody positivity) within the two risk groups with the highest prevalence: the dairy cattle farmers and veterinary professionals.

In all three models, age was included as a possible confounder and county was added as a random factor. Gender was included in the first model due to an imbalance of the gender groups within the risk groups.

All *p*-values <0.05 were considered statistically significant.

The study is in accordance with the Declaration of Helsinki and was approved by the Research Ethics Committee of the University of Tartu (licenses no. 216/T–15, 25 Jun 2012, and 235/M–26, 17 Mar 2014). Samples from the general population were collected by the EGC and covered by separate study permits. Veterinarians, ruminant farmers, and hunters signed a written consent form before the blood samples were collected by nurses. Sample tubes were labelled with an anonymous ID code and analyzed blinded. The volunteers who provided contact information were notified about their serology results. Contacts for further information were given. If individuals had medical questions, they were directed to their family physician.

## 3. Results

The test results and descriptive demographic characteristics for each study group are presented in Table 1. Out of the 84 samples retested with a confirmatory IFA test, 76 were confirmed to be positive and 8 tested negative. A total of 19 samples with negative ELISA results with OD values of 0.38 and above were found to be positive with IFA. One positive ELISA result was found to be negative with IFA. More detail information on OD values of positive and negative ELISA results and corresponding IFA results is given in Supplementary Materials, Table S1.

In Table 2, the comparison results for the prevalence within the general population and the different risk groups (model one) are presented.

The detected *C. burnetii* antibody prevalence among veterinary professionals and dairy farmers was 9.62% and 7.73%, respectively (Table 2), which were significantly higher than in the general population (3.9%). *Coxiella burnetii* antibody prevalence in the other risk groups was not statistically different from the general population, ranging from 2.27% to 3.73%.

Results of the regression analyses on the risk factors for veterinary professionals and dairy keepers are shown in Table 3 and Table 4, respectively.

## 4. Discussion

### 4.1. Methodology

Regarding the geographical distribution and gender balance, the group of randomly selected EGC donors represented the Estonian general human population well. The number of blood samples collected from all possible risk groups remained smaller than expected due to low interest. Thus, the power of the present study is not sufficient to detect all relevant associations, and the reliability of prevalence estimates may be questionable. Additionally, the diversity within the risk groups might not have been fully represented. However, a sufficient number of samples were collected to allow comparisons between study groups and identify the most significant associations. The number of individuals representing beef cattle and small ruminant keepers was relatively small, thus not much useful information could be obtained about these groups.

Blood samples from the risk groups were collected later than the general population plasma samples, which might have provided time for the infection to spread if any outbreaks would have occurred. This might have increased the *C. burnetii* antibody prevalence within the risk groups, and, therefore, the results might not be directly comparable with the general population. An increase of *C. burnetii* antibody prevalence over time in the general population has been previously shown in Denmark [7]. However, in the present study, the prevalence in all selected risk groups (e.g., hunters) was not higher than in the general population, adding confidence that the observed differences were not solely due to the temporal change in prevalence.

Based on the findings, individuals from the group of veterinarians and hunters tended to be younger (mean age 35.43 and 40.59 years, respectively) as compared to the general population (mean age 43 years), while farmers were generally a bit older (mean age >45 years). Dairy cattle farmers and veterinary professionals were predominantly female, while beef cattle farmers and hunters were predominantly male. The gender distribution of small ruminant farmers was similar to the general population. The mean ages were generally close to each other and thus comparable, while gender distribution reflected the genders generally represented in these professions and hobbies (hunting) in Estonia.

### 4.2. Prevalence

The antibody prevalence within the general adult population of Estonia detected in this study (3.9%; 2.87–5.29 CI) was somewhat lower when compared to findings from most of the other European countries. However, ELISA was used as a primary test for antibody detection in this study and only 2% of ELISA negative samples with the highest OD values were retested with the IFA test. Thus, we may have missed some positive individuals and the prevalence may be somewhat underestimated. Nevertheless, only one IFA positive sample was detected among ELISA negative samples with the lowest OD value tested, thus the number of missed positives is likely not large.

Furthermore, the study populations and laboratory methods used in prevalence studies in different countries vary, thus any comparisons between countries should be taken with caution.

Lower prevalence compared to the present study has been observed in the Netherlands in a 2006–2007 study based on random samples from the general population (2.4%) [4] and in a 2010–2015 study based on tissue and cell donor samples (2%) [59]. In contrast, in the early study from 1983, higher prevalence was observed representing the general adult population (15.2%–60.4%) [60]. The *C. burnetii* antibody prevalence in Denmark was reported to be 9.6% in 2006 and 11.1% in 2007 [7]. In Northern Ireland, 12.8% of the general population had antibodies against *C. burnetii* infection in 1986–1987 [52]. So far, no outbreaks have been detected in humans from Denmark and Northern Ireland (United Kingdom). Smaller outbreaks have been reported in France [15,38], Italy [16], Germany [13,61], Spain [17,20], Poland [18], Hungary [19], Scotland, Wales, and England (United Kingdom) [48,62,63]. These findings, and reported cases of illness [12], suggest that Q fever could be a threat to human health in endemic countries.

Based on the results of this study, *C. burnetii* infection is present in Estonia, and human infections are occurring. Since Q fever has never been reported within the people of Estonia [53], the disease in humans is likely undiagnosed. In this study, we did not identify the stage of infection in the tested individuals, and thus it remains unknown how many of the studied individuals had had a recent infection. Further studies are needed to understand the dynamics of the zoonosis in the Estonian human population subgroups.

### 4.3. Risk factor Analysis

Two occupational risk groups with a significantly higher prevalence of individuals with *C. burnetii* antibodies (veterinary professionals and dairy farmers) were identified in this study. It has been shown previously that humans with exposure to animal reservoirs have a higher risk of exposure to *C. burnetii*. In Denmark, 84% of seropositive humans had contact with cattle in their occupation [7], and the odds of being seropositive in the adult farm-occupied persons of Northern Ireland was reported to be more than five times higher when compared to non-farmers [52].

High antibody prevalence in farm-related population groups has been described by Schimmer et al., who found that 73.5% of dairy goat farmers and 71.4% of dairy cattle farmers were *C. burnetii* seropositive in the Netherlands, based on samples from 2009–2010 and 2010–2011, respectively [8,10]. Szymańska-Czerwińska et al. [11] reported that 31.12% of persons working in Polish farms tested *C. burnetii* antibody positive before 2011. The seroprevalences in risk groups were considerably higher in the Netherlands and Poland than in the present study.

Q fever outbreaks are often associated with small ruminant farming [13,19,43], but there are indications of bovine-related outbreaks also occurring. In five disease outbreaks in Poland, the description of the outbreaks and antibody findings suggest that the source of *C. burnetii* for humans were infected dairy cattle, although the shedding of bacteria by the cows was not detected [18].

In our study, the dairy cattle farmers from *C. burnetii* antibody positive herds had higher odds of being seropositive when compared to the farmers whose herds were tested negative. This is in line with the findings of Schimmer et al., who found that employment in a seropositive goat farm increased the chances of being seropositive in the Netherlands [8], but a similar relation was not observed among Dutch dairy cattle keepers, although *C. burnetii* seropositivity was significantly associated with many risk factors related to herd infection [10].

The *C. burnetii* seroprevalence among Dutch veterinarians in general was 65.1% and among livestock veterinarians was 69.2% in 2009 [9]. In Belgium, the prevalence estimates in similar study groups were, respectively, 45.4% and 58.3% in 2013 [47]. The relatively high prevalence of *C. burnetii* antibodies in Dutch and Belgian veterinarians is presumably caused by frequent contacts with infected animals [9,47]. The large Q fever outbreak in goats in the Netherlands in 2005–2010 has likely also contributed to the high seroprevalence among veterinarians [21,22]. *Coxiella burnetii* specific antibodies were found only in 4.29% of Polish veterinarians, which has been explained by a low level of contact with farmed ruminants in the group of examined veterinarians, an absence of major Q fever outbreaks in Poland, and the vaccination of animals [64].

In our study, working with farm animals increased the risk of testing *C. burnetii* antibody positive for veterinary professionals. Veterinary students practicing with living animals, particularly ruminants, and studying the specialty of farm animal medicine, have previously been described as a risk group for acquiring the infection [65,66]. US veterinarians working in animal food practice or treating ruminants, horses, poultry, or swine have also been reported to be more likely to test *C. burnetii* antibody positive [67].

Q fever is not a notifiable disease in humans and animals in Estonia, but is reportable in animals (meaning compulsory reporting by diagnostic laboratories in regular reports to veterinary authorities) [68,69], thus the disease has never been diagnosed in humans. Animal testing is carried out when there is clinical suspicion for Q fever or may be due to animal trade requirements. In the event of an outbreak in animals, the Infectious Animal Disease Control Act should be followed, but there are no specific regulations for Q fever in Estonia [70]. Since the contact with infected animals increases the risk of infection and illness in humans, national authorities ensuring public health need to be aware of the potential risks and provide prompt, clear, and accurate information on Q fever to all concerned parties when zoonosis is detected in the animal hosts. It is particularly important to inform pregnant women and other immunocompromised persons about the hazards related to working or having other contact with ruminants of unknown *C. burnetii* status. To prevent infection, unpasteurized milk should not be consumed.

Age and gender had no significant effect on the odds of testing seropositive within the studied groups. Nevertheless, age and gender were included in the regression models (gender in model 1 and age in all models) as potential confounders, as the gender and age groups were unbalanced in the studied risk groups. In other studies, it has been shown that the risk of testing antibody positive increases with age [4], as does the risk of developing acute Q fever [43] or Q fever endocarditis [71]. In contrast, in studies from Richardus et al. [60] and Schimmer et al. [8,10], the differences were not observed, suggesting that exposed individuals can acquire the infection at an early age.

The lack of difference between the genders for testing *C. burnetii* positive in this study is similar to observations by Schimmer et al. [4] and Szymańska–Czerwińska et al. [11]. However, there are studies that have observed a larger proportion of males testing *C. burnetii* antibody positive as compared to females [52,60], but in Denmark, and among Dutch risk groups, the exposure status to potential sources of infection were not identified in studies and may be different for males and females [7,9,10,47].

It has been previously shown that the geographical region of a country may play a role in the exposure to *C. burnetii* [7,22,43]. In this study, neither the county of residence nor the geographical area had an effect on seropositivity for *C. burnetii* infection of investigated persons, indicating the ubiquitous spread of the infection throughout Estonia. This can be explained by a relatively even distribution of cattle herds and animal production in general, without particular geographical differences [72].

## 5. Conclusions

There is evidence that exposure for *C. burnetii* infection is present in all of the tested risk groups and the general population and may pose a health threat.

Veterinarians, their assistants, and dairy cattle keepers have a significantly higher risk of testing seropositive, likely due to exposure from infected animals and a contaminated environment.

The presence of *C. burnetii* antibodies in bulk tank milk and frequent contact with farm animals significantly increases the odds of humans testing *C. burnetii* antibody positive, pointing to this risk group meriting further attention and having the highest potential of prevention measures. Drinking unpasteurized milk and having contact with domestic ruminants with unknown *C. burnetii* status should be avoided in all population groups, especially by pregnant women and immunocompromised persons.

## Figures and Tables

**Table 1:** Descriptive characteristics of the study groups showing *Coxiella burnetii* antibody testing results, distribution in age, and gender in different risk groups and the general population group.

Variable	General Population	Veterinary Professionals	Dairy Farmers	Beef Farmers	Small Ruminant Farmers	Hunters
Total N/n ^1^	1,000/39	156/15	181/14	41/1	44/1	134/5
Age (years)						
Range	18–86	22–63	21–78	28–67	25–76	16–74
Mean	43.1	35.4	49.9	47.0	46.9	40.6
MV ^2^	0	2	2	2	1	18
Gender n (%)						
Male	444 (44.4)	25 (16.0)	35 (19.3)	30 (73.2)	20 (45.5)	121 (90.3)
Female	556 (55.6)	131 (84.0)	146 (80.7)	11 (26.8)	24 (54.5)	13 (9.7)

^1^ Number of tested individuals (N)/number of individuals with a *C. burnetii* antibody positive result (n). ^2^ Missing values: Number of persons excluded from the statistical analysis due to not giving information about their age.

**Table 2:** Prevalence and odds of testing seropositive for *Coxiella burnetii* antibodies in the general population as compared to different risk groups using binomial mixed effects logistic regression analysis. The county was added to the model as a random factor.

Variable	Total *N*/*n*	Prevalence % (95% CI ^3^)	Odds Ratio (95% CI)	*p*-Value
General population	1000/39	3.9 (2.87–5.29)	1.00	
Veterinary professionals	156/15	9.62 (5.91–15.26)	2.68 (1.40–5.15)	0.003
Dairy farmers	181/14	7.73 (4.66–12.56)	1.92 (1.00–3.70)	0.049
Beef farmers	41/1	2.44 (0.43–12.60)	0.67 (0.09–5.02)	0.695
Small ruminant farmers	44/1	2.27 (0.40–11.81)	0.58 (0.08–4.30)	0.592
Hunters	134/5	3.73 (1.60–8.44)	0.97 (0.33–2.86)	0.960
Age (years)			1.01 (0.99–1.02)	0.411
Gender (Ref. ^4^ = male)			1.19 (0.70–2.03)	0.523

^3^ 95% confidence intervals (95% CI); ^4^ Reference (Ref.)

**Table 3:** Results of the binomial mixed effects logistic regression model for the risk factors of testing seropositive for *Coxiella burnetii* antibodies among veterinary professionals. The county is included as a random factor.

Variable	Total *N*/*n*	Prevalence % (95% CI)	Odds Ratio (95% CI)	*p*-Value
Other vet. professionals	110/7	6.36 (3.12–12.56)	1.00	
Farm animal professionals	46/8	17.39 (9.09–30.72)	3.14 (1.06–9.27)	0.038
Age (years)			1.01 (0.96–1.06)	0.704

**Table 4:** Binomial mixed effects logistic regression model for the risk factors of testing seropositive for *Coxiella burnetii* antibodies among dairy cattle farmers. The county is included as a random factor.

Variable	Total *N*/*n*	Prevalence % (95% CI)	Odds Ratio (95% CI)	*p*-Value
Dairy farmers whose BTM ^5^ tested *C.b.* ^6^ negative	73/3	4.11 (1.41–11.40)	1.00	
Dairy farmers whose BTM tested *C.b.* positive	44/8	18.18 (9.51–31.96)	5.26 (1.31–21.06)	0.019
Age (years)			1.00 (0.93–1.07)	0.989

^5^ Herd bulk tank milk (BTM) sample; ^6^ *Coxiella burnetii* (*C.b.*) antibody.

## Supplementary Materials

The following are available online at https://www.mdpi.com/2076-2607/7/12/629/s1, Figure S1: average proportions of animal patients handled by farm-animal and other veterinarians, Table S1: descriptive characteristics of *Coxiella burnetii* Phase 2 IgG ELISA and Q Fever IFA IgG test results.
