# Supplementary material for: Coxiella burnetii Antibody Prevalence and Risk Factors of Infection in the Human Population of Estonia
Source: Microorganisms. 2019 Nov 29;7(12):629. doi: 10.3390/microorganisms7120629 (PMC6956122; doi:10.3390/microorganisms7120629)
Supplement: Supplementary file 1 [file microorganisms-07-00629-s001.pdf]

Supplementary materials

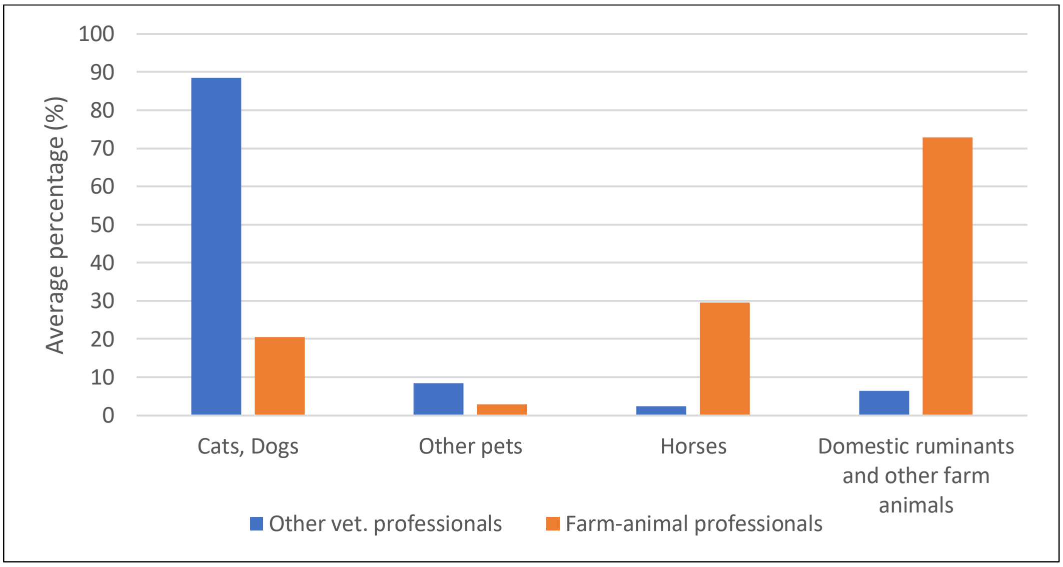

Figure S1. Average proportions of animal patients handled by farm-animal and other veterinarians.

Table S1. Descriptive characteristics of *Coxiella burnetii* Phase 2 IgG ELISA and Q Fever IFA IgG test results.

Test result	Total	Optical density (OD)			OD quartiles		
	**N**	**Min.**	**Max.**	**Mean**	**Lower**	**Upper**	**Range**
ELISA+[7]/IFA+	57	0.386	2.087	0.583	0.504	0,760	0.256
ELISA+/IFA-[8]	1	0.525		NA[9]	NA	NA	NA
ELISA-/IFA+	19	0.382	0.502	0.434	0.395	0.441	0.046
ELISA-/IFA-	7	0.382	0.447	0.397	0.390	0.406	0.016
ELISA-	1460	0.061	0.394**	0.126	0.095	0.185	0.090
ELISA-/IFA-*	12	0.051	0.091	0.069	0.059	0.085	0.026

[7] + – positive test result

[8] - – negative test result

[9] NA – not applicable

* ELISA test results with low OD value tested with IFA for inside validation

** single sample missed by mistake
